# The Perception System of Intelligent Ground Vehicles in All Weather Conditions: A Systematic Literature Review

**DOI:** 10.3390/s20226532

**Published:** 2020-11-15

**Authors:** Abdul Sajeed Mohammed, Ali Amamou, Follivi Kloutse Ayevide, Sousso Kelouwani, Kodjo Agbossou, Nadjet Zioui

**Affiliations:** Hydrogen Research Institute, Université du Québec à Trois-Rivières, Trois-Rivières, QC G9A 5H7, Canada; ali.amamou@uqtr.ca (A.A.); follivi@irh.ca (F.K.A.); kodjo.agbossou@uqtr.ca (K.A.); nadjet.zioui@uqtr.ca (N.Z.)

**Keywords:** autonomous vehicles, advanced driver assistance systems, infrared camera, lidar, road safety, radar, sensor, sensor fusion, ultrasonic sensor, weather conditions

## Abstract

Perception is a vital part of driving. Every year, the loss in visibility due to snow, fog, and rain causes serious accidents worldwide. Therefore, it is important to be aware of the impact of weather conditions on perception performance while driving on highways and urban traffic in all weather conditions. The goal of this paper is to provide a survey of sensing technologies used to detect the surrounding environment and obstacles during driving maneuvers in different weather conditions. Firstly, some important historical milestones are presented. Secondly, the state-of-the-art automated driving applications (adaptive cruise control, pedestrian collision avoidance, etc.) are introduced with a focus on all-weather activity. Thirdly, the most involved sensor technologies (radar, lidar, ultrasonic, camera, and far-infrared) employed by automated driving applications are studied. Furthermore, the difference between the current and expected states of performance is determined by the use of spider charts. As a result, a fusion perspective is proposed that can fill gaps and increase the robustness of the perception system.

## 1. Introduction

According to the National Highway Traffic Safety Administration (NHTSA), human error is the key explanation for serious road accidents, along with environmental factors such as weather conditions, which are said to lead to accidents as well. On average, more than 6.4 million automobile accidents are registered in the United States (US) annually, of which 1.561 million are weather related [1,2]. Most of these accidents occur on wet pavements (due to rain and snow), while just 3% of them occur in the presence of fog. Similarly, the relationship between road accidents and adverse weather conditions across Europe has been outlined in the COST Action TU0702 report [3]. To mitigate road car accidents, the automation of many driving functions has been successfully implemented in most of the new-generation commercial vehicles. To categorize these systems, the Society of Automobile Engineers (SAE) has defined six levels of automation, ranging from 0 (zero automatic driving maneuver) to 5 (fully autonomous navigation). While the deployment of fully autonomous vehicles (level 5) is still expected in a few years, the current commercial vehicles are equipped with the Advanced Driver Assistance Systems (ADASs), which are usually classified between SAE autonomy level 2 and level 3 [4]. In brief, the ADASs use an environment perception module consisted of several sensors whose objective is to provide relevant data necessary to interpret the surrounding scenes near the vehicle. In normal climatic conditions, the reliability and benefits of the ADASs have gained popularity and confidence [5]. However, in adverse weather conditions, the experience of the driver is required to compensate for the failure of the ADASs to appropriately perceive the surrounding environment, which usually results in severe accidents. Despite the significant effect of weather conditions on intelligent navigation perception systems, most of the review papers related to ADASs mainly focus on the efficiency of algorithms without considering climatic conditions (such as snow, sleet, rain, and fog). In fact, the capability of autonomously and robustly perceiving the surroundings in all weather conditions has not been fairly taken into consideration. Moreover, most datasets focus on urban traffic in perfect light, and clear weather conditions are often preferred for testing purposes (such as daylight and sunny weather). Just 12 of the current 36 publicly accessible databases, such as AMUSE, CCSAD, CMU, ESATS, Elektra, Heidelberg, JAAD, Oxford, Stixel, HCI, and TROM, are designed to contribute to autonomous driving under adverse conditions (night fog and rain conditions) [6]. Only a few papers have addressed the sensors and their performance in severe weather conditions. For instance, in [7], a global review of the state-of-the-art of automated vehicles is presented, including an overview of the system architecture with a briefing on key functions, such as perception, localization, planning, and control. In addition, emerging algorithms for addressing spatial information, semantic information, and target motion tracking are presented. However, the study addresses the performance of sensors under normal weather conditions but does not cover the impact of intemperate or changing weather on the performance of the sensor, which is the gap to be discussed and filled. Similarly, in [8], the review article considered one of the main functions of an autonomous vehicle, i.e., perception systems, and addressed the role of sensors such as artificial cameras, radars and lidars in the perception environment and their performance based on popular algorithms used in obtaining spatial and semantic information of targets, along with the tracking of motion. In addition, the authors provided insight into current university research centers, technology firms, and their participation in the development of autonomous driving. However, this review article only provides very general information on the impact of varying weather on perception sensors and only lidar performance issues were highlighted in selected weather conditions. In [9], a systematic review of perception systems for sensing the environment and a position estimating system with various sensors and a sensor fusion algorithm is presented. In addition, insight into model-based simulators and the current state of regulations around the world is presented. However, the challenges faced due to varying weather on perception sensors are not highlighted. It is necessary to be aware of the impact of the weather on perception when driving in all weather conditions on highways and urban transport. Improved detection in severe weather conditions will help to solve sensing problems without any further efforts in algorithm processing. Therefore, there is a need for a unifying study that combines state-of-the-art information on the effect of weather on ADASs, as well as the related perception system.

Our contribution offers information which can fill the missing gap in articles [7,8,9], related to surveys of sensing technologies and various weather impacts, on diverse sets of perception sensors (such as radar, lidar, ultrasonic, camera, and far-infrared).

- Additionally, a 3D visualization of state-of-the-art sensing technologies is shown using a spider chart, clarifying emerging trends and gaps in development. Based on the spider chart information, sensor reliability challenges and gaps can be tackled in the efficient implementation of automated driving in all weather conditions.
- Sensor fusion perspective is proposed, which involves several strategies and tasks, that will help to facilitate active sensor toggling (switching). The active sensor toggling strategy helps in the selection of sensors depending on the environment awareness context. Moreover, a potential combination of sensors is proposed for selected driving safety applications for all weather drives.

The rest of the study is organized as follows: Section 2 explains the development of an intelligent vehicle and its safety applications, focusing on the various usages of perception sensors in production. Section 3 discusses some of the well-known safety applications of ADASs and semi-autonomous vehicles with a focus on their vulnerability to extreme weather conditions. Section 4 provides a detailed overview of the sensors used in driver assistance applications, and this section highlights key points of individual sensors, like the design of construction, working principle, strategies employed by individual sensors in the perception of the environment, and the limitations of sensors in various weather environments and, finally, the section ends with a demonstration of individual sensor performance using a spider chart. Section 5 consists of a sensor fusion perspective for all-weather navigation of vehicles. In this section, we highlight strategies and tasks at the time of fusing sensors for robustness and end the section with a proposal which includes potential combinations of sensors for various ADAS applications to enhance perception and eliminate functional variations. Section 6 outlines conclusions and discussions on today’s limitations in achieving complete autonomous drives.

## 2. Evolution of Intelligent Vehicle Technology

Before analyzing the sensors technologies and their capability of navigation in difficult weather conditions, the time evolution of vehicle technology is explained. In this regard, three main phases can be observed in the literature, accounting for (i) phase I that is related to a period between 1980 and 2003; (ii) phase II that is focused on a time interval between 2003 and 2008; and (iii) phase III that started in 2008. 

### 2.1. Phase I (1980 to 2003)

During this phase, the dynamic stability of vehicles was one of the focal points. Inertial sensors incorporated into inertial measurement units (IMUs) combined with an odometer were often used to improve the stability of the vehicle, particularly when the road had several curves, and this soon led to driver assistance like anti-lock braking systems (ABSs), followed by traction control (TC) and electronic stability (ECS) [10]. Mercedes has shown efficacy and importance for human life with the combined ABS and ECS systems and the “Moose Test” has attracted public and official attention [11]. Nevertheless, safety concerns were limited to drivers and passengers, increasing concern about mobility and the safety of human life in the surrounding area, which led the way to the development of external sensors. In 1986, the European project PROMETHEUS [12] involving university research centers and transport as well as automotive companies, carried out basic studies on autonomous features ranging from collision prevention to cooperative driving to the environmental sustainability of vehicles. Within this framework, several different approaches to an intelligent transport system have been designed, implemented, and demonstrated. In 1995, the vision study laid the foundation for a research team led by Ernst Dickmann, who used the Mercedes-Benz S-Class and embarked on a journey of 1590 km from Munich (Germany) to Copenhagen (Denmark) and back, using jolting computer vision and integrated memory microprocessors optimized for parallel processing to react in real time. The result of the experiment marked the way for computer vision technology, where the vehicle, with high speeds of more than 175 km/h and with minimal human intervention, was driven autonomously 95% of the time. In the same year, in July 1995, Carnegie Mellon University’s NavLab5 traveled across the country on a “No Hands Across America” tour in which the vehicle was instrumented with a vision camera, GPS receiver gyroscope, and steering and wheel encoders. Moreover, neural networks were used to control the steering wheel, while the throttle and brakes were human controlled [13]. Later, in 1996, the University of Parma launched its ARGO project, which completed more than 2000 km of autonomous driving on public roads, using a two-camera system for road follow-up, platooning, and obstacle prevention [14]. Meanwhile, other technologies around the world have made way in the market for various semi-autonomous vehicle applications. For example, to develop car parking assistance systems, ultrasonic sensors were used to detect barriers in the surroundings. Initially, these systems had merely a warning function to help prevent collisions when moving in and out of parking spaces. Toyota introduced ultrasonic back sonar as a parking aid in the Toyota Corona in 1982 and continued its success until 1988 [15]. Later, in 1998, the Mercedes-Benz adaptive cruise control radar was introduced, and these features were initially only usable at speeds greater than 30 km/h [16]. Slowly, autonomous and semi-autonomous highway concepts emerged and major projects were announced to explore dynamic stability and obstacle detection sensors such as vision, radar, ultrasonic, differential GPS, and gyroscopes for road navigation. The navigation tasks included lane keeping, departure warning, and automatic curve warning [17,18]. Most of these projects were carried out in normal operating environments. The phase came to a halt with the National Automated Highway System Consortium [19] on the demonstration of automated driving functions and the discussion on seven specific topics related to automated vehicles: (i) driver assistance for safety, (ii) vehicle-to-vehicle communication, (iii) vehicle-to-environment communication, (iv) artificial intelligence and soft computing tools, (v) embedded high-performance hardware for sensor data processing, (vi) standards and best practices for efficient communication, and (vii) traffic analysis systems.

### 2.2. Phase II (2003 to 2008)

Several interesting projects were published in the second phase, such as the first Defense Advanced Research Projects Agency (DARPA)Grand Challenge, the second DARPA Grand Challenge, and the DARPA Urban Challenge [20,21]. These three projects and their corresponding competitions were designed to accelerate the development of intelligent navigation and control by highlighting issues such as off-road navigation, high-speed detection, and collision avoidance with surroundings (such as pedestrians, cycles, traffic lights, and signs). Besides, complex urban driving scenarios such as dense traffic and intersections were also addressed. The Grand Challenge has shown the potential of lidar sensors to perceive the environment and to create 3D projections to manage the challenging urban navigation environment. The Velodyne HDL64 [22], a 64-layer lidar, played a vital role for both the winning and the runner-up teams. During the competition, vehicles had to navigate the real environment independently for a long time (several hours). The winner of the Second Grand Challenge (Stanley, Stanford Racing Team, Stanford University) equipped his Stanley vehicle with five lidar units, a front camera, a GPS sensor, an IMU, wheel odometry, and two automotive radars. The winner of the Urban Challenge (2007) (Boss, Carnegie Mellon University Team) with its Boss vehicle featured a perception system made up of two video cameras, five radars, and 13 lidars (including a roof-mounted unit of the novel Velodyne 64HDL). The success of the Grand Challenges highlighted some important information, for example, the size of the sensors and their numbers increased significantly, leading to an increase in data acquisition density, which resulted in several researchers studying different types of fusion algorithms. Further data acquisition density studies have paved the way for the development of advanced driving maneuvers such as lane keeping and collision prevention with warning systems to help the driver avoid potential hazards. We also note that, although different challenges have been addressed in the context of competitions in urban navigation, all of them have been faced with clear weather conditions and no specific report has been provided on tests under varying climatic conditions.

### 2.3. Phase III (from 2008)

The third phase is a combination of driver assistance technology advancement and commercial development. The DARPA Challenges have strengthened partnerships between car manufacturers and the education sector and have mobilized several efforts to advance autonomous vehicles (AVs) in the automotive industry. This has involved a collaboration between General Motors and Carnegie Mellon University (Carnegie Mellon University), the Autonomous Driving Joint Research Lab, and a partnership between Volkswagen and Stanford University (Stanford University). Google’s Driverless Car initiative has introduced commercial research into autonomous cars from a university lab. In 2013, a Mercedes-Benz S-Class vehicle [23] was produced by the Karlsruhe Institute of Technology/FZI (Forschungszentrum Informatik) and Daimler R&D, which ran 100 km from Mannheim to Pforzheim (Germany) completely autonomously in a project designed to enhance safety. The vehicle, which is equipped with a single stereo vision system consisting of several new generations of long-range and short-range radar sensors, followed the historic memorial road of Bertha Benz. Phase III has focused on issues like traffic automation, cooperative driving, and intelligent road infrastructure. Among the major European Union initiatives, Highly Automated Vehicles for Intelligent Transport (HAVEIT, 2008–2011) [24,25,26] has tackled numerous driver assistance applications, such as adaptive cruise control, safety lane changing, and side monitoring. The sensor sets used in this project include a radar network, laser scanners, and ultrasonic sensors with advanced machine learning techniques as well as vehicle-to-vehicle communications systems (V2V). The results of this project developed safety architecture software for the management of smart actuators and temporary autopilot system tasks for urban traffic with data redundancy and led to successful green driving systems. Other platooning initiatives were Safe Roads for the Environment (SARTE, 2009–2012) [27], VisLab Intercontinental Autonomous Challenge (VIAC, 2010–2014) [28], the Grand Cooperative Driving Challenge, 2011 [29], and the European Truck Platooning Challenge, 2016 [30,31], which were major projects aimed at creating and testing some successful intersection driving strategies in cooperation. Global innovation, testing, and deployment of AV technology called for the adoption of standard guidelines and regulations to ensure a stable integration, which led to the introduction of SAE J3016 [4], which allows for six degrees of autonomy from 0 to 5 in all-weather situations where the navigation tasks at level 0 are managed by the driver and the computer at level 5. To respond to these regulations, the Google driverless car project began in 2009 to create the most advanced driverless car (SAE autonomy level 5) that features a 64-beam lidar rotating rooftop, creating 3D images of objects that help the car see distance and create images of objects within an impressive 200 m range. The camera mounted on the windshield helps the car see objects right in front of it and to record information about road signs and traffic lights. Four radars mounted on the front and rear bumpers of the car make it possible for the car to be aware of the vehicles in front of and behind it and to keep passengers and other motorists safe by avoiding bumps and crashes. To minimize the degree of uncertainty, GPS data are compared to the sensor map data previously collected from the aerial, which is fixed at the rear of the car and receives information on the exact location of the car and updates the internal map. An ultrasonic sensor mounted on one of the rear wheels helps keep track of movements and warn the car about obstacles in the rear. Usually, the ultrasonic sensors are used for parking assistance. Google researchers developed an infrastructure that was successfully tested over 2 million km on real roads. This technology belongs to the company Waymo [32]. Nissan’s Infiniti Q50 debuted in 2013 and became one of the company’s most powerful autonomous cars and the first to use the virtual steering column. The model has various features, such as lane changing, collision prevention, and cruise control, and is equipped with cameras, radar, and other next-generation technology. The driver does not need to handle the accelerator, brake, or steering wheel [33]. Tesla entered the course of automated driving in 2014 [34], with all its vehicles equipped with a monocular camera and an automotive radar that enabled autopilot level 2–3 functionality. In 2018, Mobileye, focusing on a vision-only approach to automated driving, presented an automated Ford demo with only 12 small, fully automated mono-cameras [35]. Beside the projects, there are many pilot projects in almost all G7 countries to improve the introduction rate of the ultimate driverless vehicle. Moreover, the ADAS has achieved high technology readiness, and many car manufacturers are now deploying this technology in their mass-market vehicles. Although several key elements for automatic maneuvers have been successfully tested, the features are not fully covered under all weather conditions. In the next section, we discuss the various applications of ADASs which are currently available in the market and their limitations in performing in various weather conditions.

## 3. Automated Navigation Features in Difficult Weather Conditions

### 3.1. Forward Assistance

#### 3.1.1. Adaptive Cruise Control (ACC)

The ACC system helps the driver to longitudinally control the vehicle dynamics [36]. The main motivation for the ACC development is to relieve the driver from driving stress, distracting tasks, human error due to constant monitoring of speed, and maintaining proper progress in irregular traffic. This ACC feature is a combination of the cruise control with collision avoidance control. The vehicle speed is modulated based on the distance from the front vehicle (leading vehicle) [36,37,38], which is mainly intended for highway environments. Mitsubishi and Toyota introduced the cruise control function in Japan in 1996, based on lidar technologies [39]. Later, the ACC was expanded in Europe by Mercedes-Benz in 1999, where it used radar technology combined with an automatic braking system. Detecting other vehicles from a moving vehicle is a challenging task for smart vehicles in heavy traffic and winter conditions (for example, snowfall and icy roads). Particularly, the feature must deal with the vehicle skidding and sliding to avoid any collision when the road is wet, snowy, and icy. Indeed, snowfall can accumulate in the sensor locations and reduce the sensor capability to correctly perceive its surroundings. Additionally, the rainwater causes the oil and grease to rise to the top of the water on the road and creates a slippery or icy road. The popular sensors for ACC applications include the visible spectrum camera, the ultrasonic sensor, lidar, radar, and passive far-infrared cameras. The cameras can be used to know the surroundings and target vehicles ahead. However, in rain and fog conditions, sensing vehicles around and ahead becomes difficult as the lidar can send false obstacle detection alerts. Ultrasonic sensors work well in close range due to low noise in the reflection of sound waves from the targeted object. Radars are robust in all climatic conditions, however, due to their narrow detection field of view, other existing vehicles in the lane of the host vehicle cannot be adequately recognized, which may lead to a sudden collision. Passive far-infrared cameras can come in handy for adverse weather conditions. Indeed, the sensors with robust algorithms and image learning can detect obstacles accurately and robustly in most of the difficult climatic conditions due to their ability to see through fog, rain, and even snow [40,41].

#### 3.1.2. Forward Collision Avoidance (FCA)

Various studies (such as the European Collision Support Initiative) in connection with a forward collision accident technology have demonstrated that many drivers do not brake or use the full braking system capacity when facing road emergencies. This feature mainly relies on continuous monitoring from different types of sensors like cameras, ultrasonic, radar, and lidar. FCA is intended to help the driver to respond quickly and safely to any obstacle on the road. In challenging road conditions, traction is even harder to control. According to ISO 15623 2013 [42,43], the minimum distance for vehicle detection must be over 45 m. For a relative velocity of 20 m/s, azimuth lateral sensing with a single sensor should be between 9° and 18° to recognize a vehicle as wide as 1.80 m. Furthermore, it should have a sufficiently large vertical visual range (elevation) to detect a target with a height of 1.1 m. For normal weather conditions, most sensors (radar, lidar, and cameras) meet the standards, but in more difficult environments (rain and snow with low visibility), sensors face challenges to detect relevant objects. As discussed in the ACC application, lidar, camera, and ultrasonic sensor capabilities are limited in bad weather conditions, while radar can still be more robust.

#### 3.1.3. Road and Traffic Sign Recognition

Typically, road and traffic signs are either on the roadside or above the road. They give drivers important information to guide, warn, or regulate their behavior to make driving safer and easier. Road and traffic signs have special colors and symmetrical shapes, such as triangles, circles, octagons, diamonds, and rectangles. A sign’s shape, color, and its related ideogram are designed to draw the driver’s attention. Bad weather can decrease the visibility of traffic signs and an obstacle (such as a pedestrian or another vehicle) can partially obscure road signs, which may cause drivers to miss important road signs. Therefore, traffic and sign recognition technologies must be good at classifying them, even in non-ideal conditions. Radar and ultrasonic sensors cannot recognize and classify signboards. Lidar is good at mapping the surroundings, but it has a low color contrast and its elevation angle is not good enough to recognize and classify signboards [44]. For this application, machine learning with Complementary Metal Oxide Semiconductor (CMOS) cameras, have a good performance with low-expense solutions. Traffic sign recognition algorithms usually have two steps of detection and classification [45,46]. At night, cameras go blind and depend only on car headlights. However, this does not affect the performance since special paints are used on signboards make them bright at night [47]. In heavy rain and snowfall, the camera can have trouble detecting the signboards due to visibility issues. A passive far-infrared (FIR) camera works by evaluating an object’s thermal signature and emissivity, and it cannot see colors in detail. However, using FIR, which can efficiently detect and recognize signs from the background, in combination with a CMOS camera, can provide a hybrid perception solution that is robust in difficult weather conditions.

#### 3.1.4. Traffic Jam Assist (TJA)

Traffic jams are the main reasons for the development of the traffic jam assist (TJA) feature. TJA utilizes functions of ACC and lane assist (see Section 3.2) to enable convenient and safe stop-and-go driving. The TJA system responds to other vehicles by adjusting the safe distance and autonomously handles steering in a lateral direction if any free space is detected [48]. If the vehicle with TJA encounters a situation where there are too many nearby activities, such as frequent changes in adjacent lanes, lots of obstacles, and unpredictable speeds of other vehicles, the driver receives a take-over prompt [49]. TJA combines longitudinal and lateral direction monitoring to locate surrounding vehicles. Information about both lane structures, such as markings and other vehicles in the immediate environment, is needed to design the TJA function. This implies that sensors should be sufficiently capable of locating immediate surroundings at a shorter distance to the host vehicle. Environmental effects such as snow, rain, and fog have limited effects on sensor performance since the detection must be performed in traffic in a small perimeter [48]. Various experiments have shown that although the performance of sensors degrades at long ranges in different weather conditions, it is good for shorter distances. Passive far-infrared cameras are computationally challenging for short ranges. Lidar can effectively locate the surroundings. Ultrasonic sensors are very effective in short ranges with low noise. They are also very cheap compared to other sensors. Their accuracy of detection may vary since their waves are influenced by temperature due to the emitted heat of surrounding vehicles. Mid-range radars are inexpensive and effective in detecting the surroundings. For the lateral direction, cameras are a cost-effective solution for detecting lane markings and boundaries.

### 3.2. Lateral Assistance

#### 3.2.1. Lane Departure Warning (LDW) and Lane Keeping Assistance (LKA)

Unintentional lane departures on highways lead to dangerous accidents, based on various accident statistics [50]. To overcome this problem, lateral assistance (LA) has been developed. Lane departure warning (LDW) and lane keeping assistance (LKA) are two popular features. LDW warns drivers about deviation from the lane and LKA assists them with keeping the vehicle on track by controlling and steering the vehicle. For LKA systems, it is important to know the vehicle position with respect to the lane of travel. Lane detection and lane tracking are therefore critical tasks. Lane marks can sometimes be difficult to recognize on various road types due to snow, other vehicles, and changes to the road surface itself [51,52]. In all weather conditions, the lane sensing system should be capable of determining every type of markings on roads so the position and the trajectory of a vehicle regarding the lane can be reliably estimated. Since the detection of lanes does not require kinetic and shape information, cameras and lidar are mostly used [53,54]. However, difficult weather conditions and low visibility issues are still the major concerns that limit the performance of cameras. 

#### 3.2.2. Lane Change Assistance (LCA)/Blind Spot Monitoring (BSM)

Assisting drivers in changing lanes, where possible mistakes can be minimized, is the purpose of the lane change assistance (LCA) system. In urban, rural, and highway roads, the monitoring of the side lane traffic is very important for lane changing. Based on ISO standard 17387 [55], LCA tries to accomplish blind spot detection (BSD) that focuses on the vehicle approaching from behind in the side lane. For LCA, it is important to provide the driver with information about the immediate vehicles near the host vehicle. For blind spot monitoring, sensors with a medium range between 70 m and 100 m are used. Rear cameras can deliver information on the position of the vehicle approaching from behind at the time of the departure. However, the operating range is influenced by climate conditions. Passive far-infrared cameras and mid-range radar sensors can overcome weather conditions by robustly detecting surrounding vehicles in all weather conditions. Nevertheless, the cone-shaped spectrum of radar detection remains a difficult problem at bends [56]. In addition to radars, ultrasonic sensors can be used as a backup.

## 4. Sensors

### 4.1. Overview

**Figure 1:**
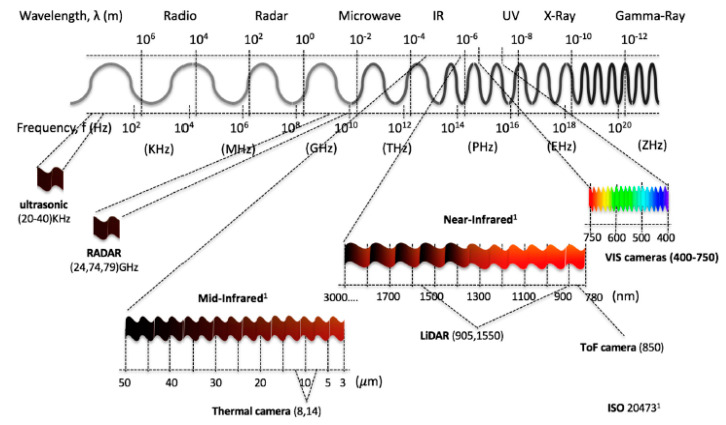
Electromagnetic spectrum according to ISO 20473 [57].

All the above features were designed to improve vehicle safety and rely deeply on sensor data. Data accuracy from the sensors depends on environmental stimuli to perceive the surrounding scene. Car sensors are classified as proprioceptive or exteroceptive. Proprioceptive sensors help measure vehicle ego movement and dynamics. Some of the proprioceptive sensors are wheel speed sensors, torque sensors, steering angle sensors, and IMUs. On the other hand, exteroceptive sensors work by detecting obstacles, and recognizing the navigation scene and help gather information from the surroundings and improve knowledge of the vehicle’s location. Exteroceptive sensors are further categorized into passive and active sensors. Active sensors include ultrasonic, radar, and lidar, which function by emitting energy in the form of electromagnetic waves or radiation and measure return time to determine parameters such as distance and position. Instead of transmitting external signals or disruptions, passive sensors (infrared cameras) receive electromagnetic waves or radiation in the environment. Active sensors are preferred to passive ones, since they have less trouble discriminating between useful data and irrelevant signals. Figure 1 shows a wide range of the electromagnetic spectrum described by ISO 20473, including the wavelength of the following sensors: CMOS camera, near-infrared sensors, lidar, thermal camera, radar, and ultrasonic sensor.

Since different environmental stimuli can include any mixture of these wavelengths, the robustness of perception can be improved by combining different sensors with nonoverlapping wavelength intervals. However, the wavelength is not the single dominant function, additional geometry (shape), spatial resolution, and accuracy must be considered to overcome surrounding scene complexity and can require advanced filters to eliminate disturbances and interference while perceiving. In the following section, exteroceptive sensors responsible for environmental perception are addressed briefly and, with findings from reviewed experimental tests, the individual sensors’ advantages and disadvantages, along with their performance capabilities in different environments, are presented and the same is demonstrated on a spider chart for visualization. All the criteria behind the spider chart aim to demonstrate the state-of-the-art performance of individual sensors, compare their capabilities with other sensors, and give an idea of individual limitations. The information used to construct spider charts was obtained from manufacturers and the evaluation of experts’ key findings, and the related references are cited in each section, describing the individual sensors. Criteria chosen for sensor information are based on the ability of the sensor to obtain target spatial information (such as location, velocity, range, and shape) and detect and recognize objects (pedestrians, cars, trees, and streetlights). These criteria help to provide information such as sensor resolution and contrast. In the same way, the individual sensor capacity to work in the precipitate and aerosol environment has been highlighted with various experimental studies. The following criteria have been used in the visualization of the spider charts, and Table 1 helps in reading and interpreting the spider chart.

**Range:** Information is gathered from various sensor manufacturers and used to describe the performance of the sensors. For example, the ability of a sensor to detect any object over 100 m and above is assumed to represent high performing sensor and is represented with Index 4 (i = 4) on the spider charts to visualize this. However, if a sensor cannot detect objects at 100 m, the output of the sensor is considered as low performing, indicated with Index 1 (i = 1). For ADAS safety driving applications, range requirements may vary.

**Resolution:** Information on resolution is gathered from noted experimental studies, whose references are cited under individual sensor performance reviews. A high performance is considered for sensor if each measurement axis of the sensor can achieve a space resolution of less than 10 cm (i = 4) or is considered a low performance sensor otherwise (i = 1). Mapping resolution helps to estimate the sensors’ ability to provide information about the position, velocity, size, and shape of the target.

**Contrast:** Information on contrast is gathered from noted experimental studies, whose references are cited under individual sensor performance reviews. If the sensor can accurately identify an object when the ambient contrast is small, then sensor performance is assumed to be high (i = 4). Furthermore, if the sensor can reliably detect an object only when the ambient contrast is very high, then the sensor is assumed to have low performance (i = 1). This differentiation helps in understanding the ability of sensors to classify and track the target of interest.

**Weather:** If the sensors can reliably perform successful detection under harsh weather conditions, it is assumed as a high-performance sensor (i = 4). Similarly, if sensor provides good detection only in clear weather conditions, then the sensor is assumed to have low performance (i = 1). The information about the weather has been gathered based on noted experimental analysis and the related references are cited under individual sensor performance reviews.

**Cost:** Details on the individual costs of the sensors were collected from various online sources. For instance, we have a unit price of automotive ultrasonic sensors ranging from USD 16 to USD 40 [58,59] and of automotive radar ranging between USD 50 and USD 220, based on the application for short–medium–long range [60]. The automotive lidar price ranges between USD 500 to USD 75,000, based on the design and configuration of lidar and, in 2020, Velodyne announced it would be introducing a USD 100 lidar sensor applicable for use in cars [61,62]. The automotive mono-camera price ranges between USD 100 and USD 1000 [63,64] and automotive thermal cameras cost around USD 700 to USD 3000 [65]. In order to visualize the sensors’ cost on a spider chart, we compared the actual price with the ideal price, as shown in Table 2. The ideal price is proposed by authors and depends on the actual price versus the acceptable price by considering the sensor design and complexity.

The following sub-section presents various sensor reviews, which highlights key points such as market penetration, the working concepts and techniques of a specific sensor in environmental perception, and the advantages and disadvantages of sensors under different weather conditions.

### 4.2. Radar

**Figure 2:**
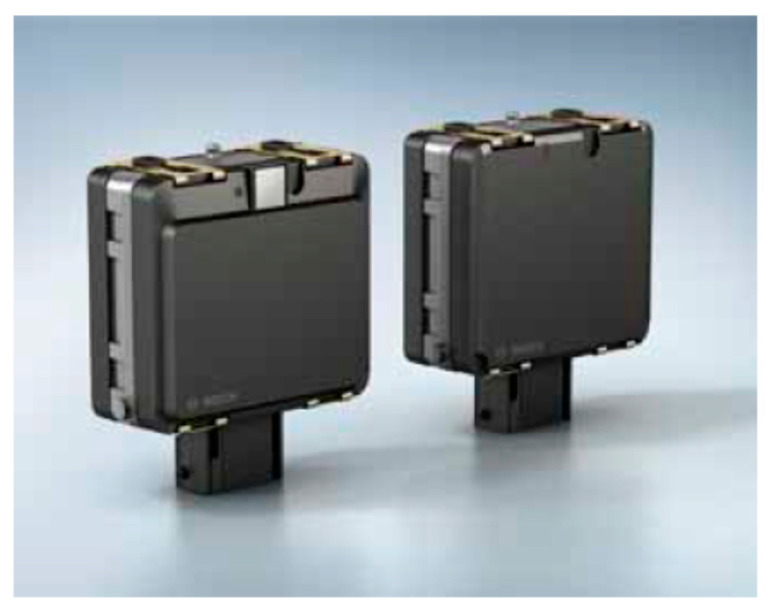
Mid-range radar from Bosch [68].

**Market penetration:** Radio detection and ranging (radar) provides smart vehicles with effective safety procedures. This sensing technology started with Hertz and Hülsmeyer’s electromagnetic wave reflection studies [66], and now has steadily improved from basic blind spot detectors and cruise control systems to semi-autonomous obstacle detection and braking functions [16]. Bosch, with its partnership with Infineon, is the dominant manufacturer of radar in the market. The other big players are Continental, Autoliv, Delphi, Elesys, Hella, Fujistu Ten, Mitsubishi Electric, ZF-TRW, SmartMicro, Denso, Valeo, Hitachi Automotive, Clarion, etc. Technical information on radar by various manufacturers is presented in [67]. In Figure 2, an image of radars from Bosch is presented. 

**Working principle:** The vehicle radar setup includes a transmitter, an antenna, a receiver, and a processing unit. The radar transmits electromagnetic waves produced by the transmitter in a known direction. If an obstacle or surface intercepts waves, they are reflected to the receiver system. The processing unit uses the captured signal to define target range, angle, and velocity. Based on applications, automotive radar sensors are categorized as short-range radar (SRR) (up to 30 m), medium-range radar (MRR), and long-range radar (LRR) (up to 250 m) [69,70,71,72,73,74,75,76,77]. Automotive radar systems typically run between 24 GHz and 76 GHz portions of the electromagnetic spectrum. In [78], an experimental distinction was made between 76 GHz operating with 4 GHz bandwidth and 24 GHz operating at 200 MHz bandwidth, with results concluding that low-bandwidth radars could not distinguish between two distinct obstacles and would send the driver or autonomous vehicle incorrect information. Many of today’s automotive radars are built on a frequency-modulated continuous wave (FMCW), because it enables easy modulation, high average power, high bandwidth, and excellent range resolution [79]. In the same reference [79], a brief review of the key developments in radar and signal processing techniques applied to the estimation of significant target parameters, such as range, velocity, and direction, are presented with mathematical illustrations. Thanks to extensive use in the automotive industry for various applications and due to advances in signal processing by applying machine learning, pattern recognition techniques, and robust algorithm developments [80], radar data now have more knowledge of object dimension [81], object orientation, motion prediction [82], and classification information [83,84,85]. 

**Degradation of radar performance:** Nonetheless, the radar performance in various weather conditions is not as good as in clear weather. Influences of adverse climates, such as precipitate and aerosol environments, on radar output were analyzed and it was concluded that precipitate environments, such as rain, had the most impact, causing a reduced range of radar, and a similar effect was found in the presence of wet snowfall. Experimental analysis in [86,87,88] supports the argument which focuses on radar performance in the rain and provides details on attenuation and backscatter effects responsible for the deterioration of radar performance in the rain. The attenuation effects that lead to a decline of the radar range are higher at a higher frequency (about a 45–50 percent decrease in the heavy and very heavy rainfall range), while backscatter effects in radar increase the noise in the receiver. It is noted that noise is high at a low frequency and causes false-positive errors at the receiver. Similarly, in [89], wet and dry snowfall impact on radar at 77 and 300 GHz frequencies was studied. The findings concluded that attenuation due to snow contributed to a decreased range of about 12~18.5 dB/km, and the study also noted that the attenuation varied greatly with snow water content. In [90], a mathematical model is presented to test wet snowfall output; the analysis shows that radar output has a similar effect on snowfall to that of rainfall. Although radar performance in precipitate surroundings has been observed to degrade, the study in [91,92], compares radar, lidar, and camera performances in simulated and real-world adverse climatic environments and concludes that radar outperforms lidar and cameras under the influence of rain. In [93,94], the effect of aerosols upon the transmission of radar signals has been investigated in controlled environments and mining applications, and the results show that radar is not impacted by the presence of airborne particles, such as dust and smoke because of its wavelengths, which are much larger than the characteristic dimensions of dust. Although radar may be a great option for all weather conditions, signal interference is still a matter of concern. More detailed information on radar interferences is presented in [95], which discusses the interference impact on radar, its characteristics, and mitigation strategies. Based on the review analysis, the advantages and disadvantages of radar are summarized and listed in Table 3. 

**Figure 3:**
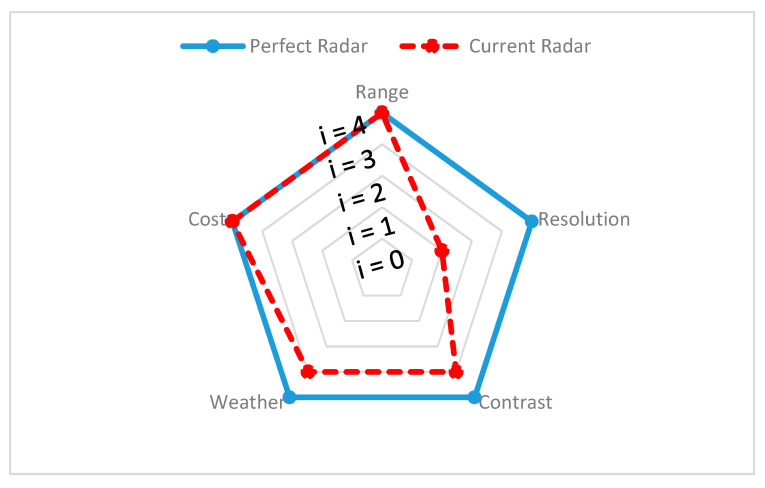
Performance gap for radar sensor based on the scores gathered.

The spider chart for radar is presented in Figure 3, where the ideal radar feature is shown with the blue bold curve, whereas the actual radar sensor output is shown with the red dashed line. The difference between both curves represents the difference between the current and desired radar sensor output.

### 4.3. Lidar

**Figure 4:**
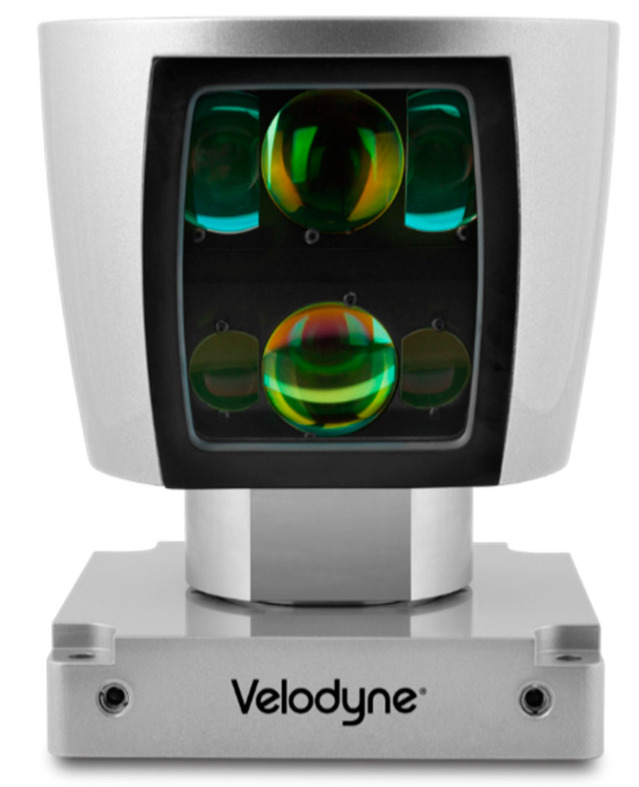
Velodyne HDL 64 lidar [99].

**Market penetration:** Lidar is the abbreviation of light detection and ranging. Lidar was developed in 1960 for the study of environmental measurements (atmospheric and oceanographic parameters) and later was developed for topographical 3D mapping applications in the mid-1990s [96]. In 2005, lidar was applied to vehicles to locate and avoid obstacles in the DARPA challenge [97]. Lidar commercial manufacturing companies include Velodyne, Quanergy, Leddartech, Ibeo, etc. The technological description of individual sensors is provided in [98]. In Figure 4, an image of an automotive lidar is shown.

**Working principle:** Just like radars use time of flight of radio wavelengths to collect target information, photodiodes are used by lidar to transmit the light pulse to the target. The optical receiver lens in the lidar system is used as a telescope for the processing of photodiode fragments of light photons. The collected reflections include 3D point clouds corresponding to the scanned environments and the strength of the reflected laser energies provides information about the range, speed, and direction of the target. Lidar manufacturers use two wavelengths: 905 nm and 1550 nm. The former is a common option for automotive manufacturers due to its reliability, eye protection, and cost-effectiveness with silicone detectors. In [100,101], an authoritative analysis on lidar, its waveform, and market penetration strategies are discussed. Lidars provide a good physical description of the target and, due to that, lidars have been used for target detection, tracking, and motion prediction., Filtering of the ground and clustering of the target [102,103,104,105] are two methods widely used by lidar for object detection, which provide the spatial information of the target. To classify and recognize objects (like pedestrians, trees, or vehicles), lidars make use of techniques such as machine learning based on object recognition [106,107,108,109], and additional methods such as global and local extraction of features to help in providing the structure of the target. Lidar uses the Bayesian filtering framework and data association methods for target tracking and motion prediction to provide information, such as velocity, trajectory, and object positioning [110,111,112]. In contrast to radar-based multi-object tracking, in which all detections are typically represented as points, lidar-based multi-tracking provides detection patterns of targets and this property of lidar scanning causes users to opt for lidar. 

**Figure 5:**
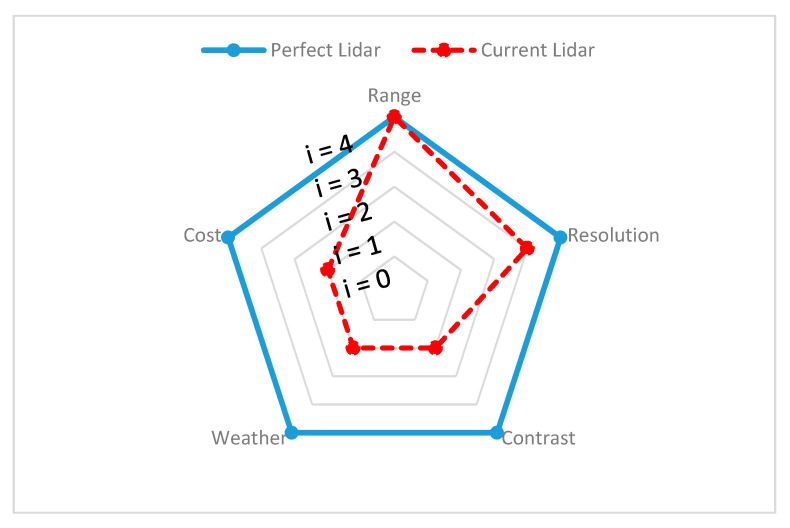
The performance gaps in lidar sensors are illustrated using a spider chart, based on performance scores.

**Degradation of lidar performance:** Lidar performance in extreme weather conditions is not as strong as expected. Adverse weather conditions increase the transmission loss and decrease the reflectivity of the target. Under the perception category (fog, snow, and rain), fog has been found to have the greatest impact on the ability of lidar, due to its high expansion and backscattering properties, which are greater than in weather conditions like snowfall and rain [113]. The challenge in fog conditions is that many transmitted signals are lost, resulting in reduced power. Reduced power would alter the signal-to-noise ratio of the lidar sensor and influence its detection threshold, which leads to degraded perception performance. In [114], the depth of lidar performance in fog is studied and the observed light is scattered by fog particles, which not only reduces the detection range dramatically, but also leads to false detections. In the same study, the fog condition showed similar performance degradation to the airborne environment. In [115,116,117], qualitative and quantitative experimental studies of the fog effect on lidar in controlled environments outlined the loss of transmission phenomena leading to low received laser power and low target visibility. Lidar scanner capacity testing has been carried out in the northern part of Finland at Sodankylä Airport [118], where fog creates a special problem. Results indicate that fog reduces the sensor range by 25%. In [119], the quantitative performance of lidar with varying rain intensity, with the help of a mathematical model, is presented and the results show that as rainfall intensity increases, the lidar cloud density is affected, increasing false-positive errors. The same effect is presented in [120], where the authors’ analysis showed that the varying intensities, size, and shape of raindrops drastically influence the attenuation rates of lidar. The effect of snow on lidar performances, such as reflectivity and propagation through the snowy environment, is evaluated in [121], using four lidars of different manufacturers. The results observed from this experiment highlight that the receiving power levels generated by snowflakes or water droplets were high (due to false-positive errors) and tended to overload the optical receiver chain. The lidar performances are also altered by airborne particles, such as dust, which have greater characteristic wavelengths than lidar. These particles prevent the sensor from imaging its surroundings, resulting in reduced visibility and incomplete target information. In another work [122], the experiment outlines that the dust particles in the air are very often detected by laser sensors and hide obstacles behind the cloud of dust. To address these limitations, the use of lidars with a 1550 nm wavelength with a strong propagation ability is suggested. However, this solution is of limited use, because of constraints such as high cost and high energy usage. The loss of efficiency due to adverse environmental conditions was examined in [123], and a comparison was made between lidars with wavelengths 905 nm and 1550 nm. The findings of [123] have also shown that lasers with a wavelength of 1550 nm have a much higher water absorption compared to 905 nm lidar. Furthermore, due to the advantage of the higher wavelength (1550 nm), higher power can be used for the transmission of lidar signals, which could result in an increased range of detection in adverse climatic conditions, while maintaining eye safety regulations. The advantages and disadvantages of lidar are outlined in Table 3. The ideal lidar function is shown in Figure 5, with the blue bold curve, while the current lidar sensor performance is shown with the red dashed line. The difference between both curves reflects the gap between current and desired lidar sensor performance.

### 4.4. Ultrasonic Sensor

**Figure 6:**
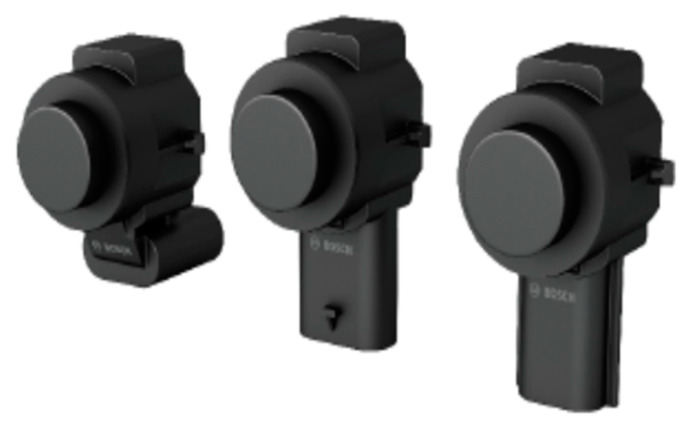
Ultrasonic surround sensor from Bosch [126].

**Market penetration:** It is a very difficult task for drivers to track vehicles on the road, because they cannot always be aware of the presence of all obstacles around the vehicle. The ultrasonic sensor is popularly used to measure proximity to obstacles in a very short range and is widely used in areas where distance- and occupancy-related detections are needed. For vehicle applications, the popular uses of ultrasonic sensors include (1) low-speed car parking and (2) high-speed blind spot detection. For example, in the consumer market, Tesla Motors dominates the use of ultrasonic sensors and has already used ultrasonic sensors for some of its functions, such as Tesla’s advanced parking assistance with the “Auto-park” and “Summon” features, which promote the self-driving of a vehicle with a driver outside and the monitoring of a blind spot at high speed. The “Autopilot” and “Autosteer” features monitor surroundings of the vehicle and stabilize vehicle heading accordingly [124]. As far as parking sensors are concerned, more than half of the new vehicles in Europe and Asia have rear parking sensors, so it is not surprising that the global market for car parking sensors is projected to grow steadily over the next few years, with a compound annual growth rate of almost 24 percent by 2020 [125]. Currently, Bosch is the principal manufacturer of ultrasonic sensors and the technical specifications of the ultrasonic sensor are presented in [126]. In addition, an image of automotive ultrasonic sensors is shown in Figure 6.

**Working principle:** The configuration of the ultrasonic sensor consists of a piezoelectric material transducer, charged with an alternating electrical voltage, which causes fluctuation and short sound wave bursts. This sound wave is transmitted to the target, which reflects the sound of the sensor. The reflective sound echo of the sensor provides information on the distance, velocity, and angle of the obstacle. Information such as the distance to the target can be calculated by the flight technique, the speed can be estimated by the Doppler shifting process, and the target direction can be determined by the strength of the reflected sound wave [127,128,129]. Ultrasound speed is easily influenced by factors such as temperature, humidity, and wind, which cause the sound pressure to decay exponentially with the spread of sound over a distance, resulting in significant effects on the accuracy of the measurement and complicating the study of ultrasound sensors, which is why it is important to check the temperature and other transmission factors [130]. In addition to the speed of sound, the ultrasonic sensor accuracy also depends heavily on the reflective characteristics of the target surface, such as curvature, terrain, and design target material [131]. The ratio of humidity of the air is important for the determination of the maximum range of sensors. Frequencies greater than 50 kHz will result in weaker echoes due to the attenuation of airborne sounds, while the proportion of interference sounds at the receiver is higher for frequencies lower than 40 kHz. Due to this limitation, ultrasonic sensors on vehicles typically operate within a frequency band of between 40 and 50 kHz, which has been shown to be the best trade-off between acoustic performance (sensitivity and range) and ambient noise resistance [131,132,133,134]. A brief review of the state-of-the-art ultrasonic sensor with wave propagation, atmospheric attenuation, sound wave reflection, and target tracking, along with market penetration of ultrasonic sensors, is presented in [135]. Most of the studies on ultrasonic sensor performance are presented in [136,137,138,139,140,141,142,143,144,145,146,147], which focus on the ability of sound waves to detect, reflect, and track the target. Besides, sound wave propagation in the presence of changing winds and temperatures, as well as several new designs to enhance resolution, have been introduced. However, the results of all experiments show that good resolution is achieved within a shorter timeframe and the target wave reflection is accurate and reliable. The authors of [136,137,138,139,140,141,142,143,144,145,146,147], suggest the use of ultrasonic sensors for near-field perception based on their experimental studies. 

**Figure 7:**
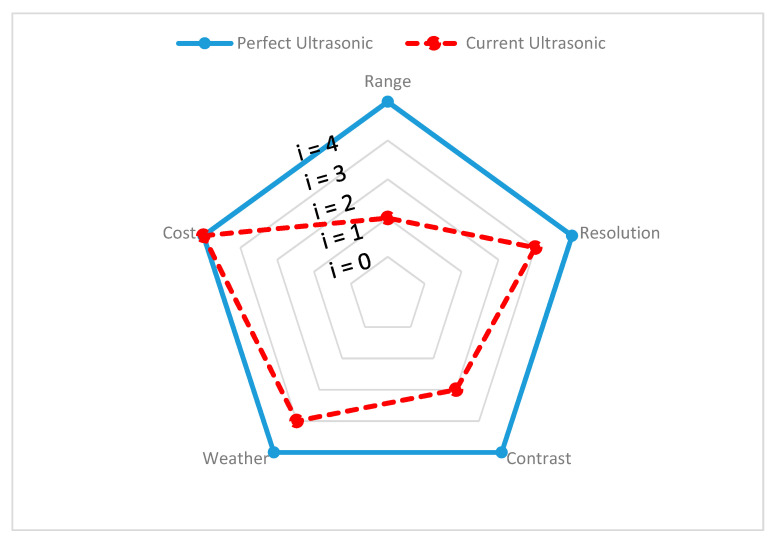
Ultrasonic sensor spider chart, showing performance gap.

**Performance degradation of ultrasonic sensors:** As the ultrasonic wave spreads through a homogeneous gas such as air, absorption and dispersion combine to give the overall attenuation level. Precipitation (fog, snow, and rain) and the presence of airborne particles have an insignificant effect on sound waves, although precipitation clearly affects humidity and may also affect wind and temperature gradients. Under normal circumstances, atmospheric absorption may be neglected, except where long distances or very high frequencies are involved [148,149,150]. Although a low-cost, high-performance ultrasonic sensor appears to be an appropriate choice for all-weather perception, it lacks safety and can be easily spoofed. For example, in [151], ultrasonic sensor vulnerabilities and their impact on performance have been exposed. The safety of the ultrasonic sensor, in [152] has also been analyzed and the study outlines the reliability of ultrasonic sensors for future use. Based on the gathered information, the current performance (red dashed lines) and desired performance (bold blue line) of ultrasonic sensors are presented in Figure 7, and Table 3 highlights ultrasonic sensor advantages and disadvantages.

### 4.5. Vision-Based Systems

**Figure 8:**
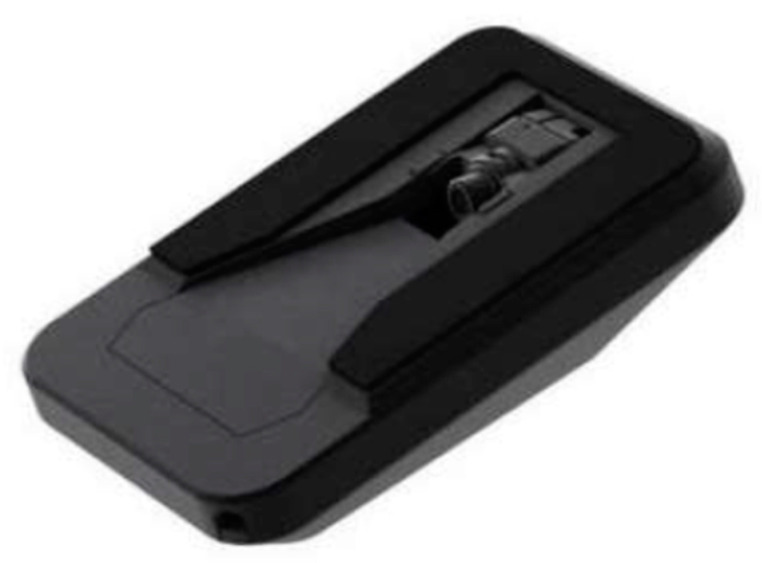
Intelligent mono-camera by Mobileye [154].

**Market penetration:** Human driving is primarily based on an analysis of the characteristics of the surrounding vehicles, including obstacles and road signs. A camera provides a way to obtain some of this information for automated operation. Almost all SAE degrees of autonomy greater than 1 use cameras. Cameras are the only kind of imaging equipment capable of seeing colors. In Figure 8, a mono-camera from Mobileye is shown. Mobileye is a popular manufacturer currently leading monovision with smart technology and technical specifications of the intelligent vision-based camera can be found in [153].

**Working principle:** The camera is a digital lens imagery system that works by collecting and translating the image of an object into electrons on a pixel image sensor. Later, the camera capacitors convert electrons into voltages, which are later converted into an electronic digital signal [155]. Two imaging sensors, the charging coupling device (CCD) and the complementary metal oxide semiconductor device (CMOS-D), are typically used in real-time applications. Brief comparisons between the CCD and CMOS-D can be found in [155,156]. CCD cameras deliver excellent low-noise performance but are expensive and, as an alternative, the CMOS-D has been developed to reduce production costs and power consumption. Because of this advantage, the CMOS-D is widely preferred for automotive applications in the related industry. Cameras, in conjunction with computer vision and deep learning techniques, offer environmental information, such as detection of the target, their related physical descriptions (like the position of moving targets, size, and shape) and semantic descriptions (like recognizing and classifying trees, vehicles, traffic lights, and pedestrians). Camera information is easy to understand, which makes it more popular than other sensors. Various configurations exist in the camera and out of different configurations, monocular and stereo vision camera solutions are a common choice for researchers. In monocular systems, only one camera is used to detect, track, and measure longitudinal distances, based on landscape geometry. There is a downside to distance measurement in the monocular camera, since the distance is measured by using the position of the pixel in the vertical direction of the given image coordinates, which typically results in errors, due to the lack of direct depth measurements for the captured images. Compared to monocular cameras, stereo cameras with two cameras have an additional feature for measuring the distance between objects. Using stereo-view-based methods, two cameras can estimate the 3D coordinates of an object. A brief analysis of techniques for real-time obstacle detection and classification linked with various algorithms using the stereo camera is presented in [157]. While stereo vision cameras are effective in target detection and classification, they are more expensive than mono-cameras, and they also have problems with calibration and computational complexity. One of the reasons why vision-based approaches are favored in urban traffic is the identification of traffic lights. Traffic light detection methods in cameras are based on image processing, machine learning, and map-based techniques. Within an image-processing procedure, a single or multiple thresholding, filtering, and extraction operations are performed on an image to obtain a particular result. A slight miscalculation can influence image efficiency, which can be addressed by machine-based learning methods and processing algorithms. Nonetheless, to achieve optimal output using machine learning methods, it is important to collect massive training datasets and train the model for a significant amount of time. Map-based methods are used to overcome this limitation [158]. In [159], an image processing method used by a vision system for traffic light detection and recognition is presented, which involves image modifications like RGB to Hue Saturation Value (HSV) conversion and filtering. Furthermore, the article [160], proposes a system based on a fast convolutional neural network (CNN) based on the YOLOv2 network. This algorithm can detect the location of a traffic sign and classify it according to its form. Deep learning approaches are also provided in [161], which outperform image processing methods for the robust detection and recognition of traffic lights. 

**Figure 9:**
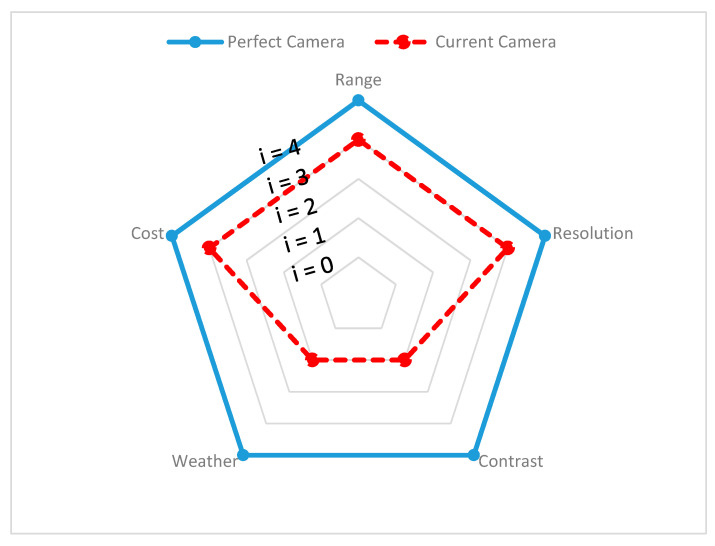
Camera sensor spider chart. The difference between the red dashed line and the solid blue line is the performance gap.

**Vision-based system performance degradation:** While advanced methods have improved recognition techniques, small variations in weather still influence camera measurements. The camera is very sensitive when faced with adverse climatic conditions. A camera in an aerosol environment experiences decreased visibility and contrast, and is unreliable in object recognition, and a camera is not recommended for environmental detection and vehicle control tasks under foggy conditions, as per [162]. In the same reference [162], a full description of the rain and fog interactions with a camera is also provided. Camera sensors have an advantage over object detection and classification and are important for automated safety systems. However, in [163], an indoor rain simulator was used to systematically investigate the effects of rain on camera data and the results outline that the performances of the camera sensors were mainly affected by decreased gradient magnitudes, resulting in a shift in the location and size of the bounding boxes during the detection process, leading to a decline in classification scores and resulting in uncertainty. Similarly, based on indoor experiments [164], the authors experimentally researched the effects of rain and showed that raindrops lead to an increase in the average intensity of the image and a decrease in contrast. The study presented in [165], develops an approach to quantifying the vulnerabilities of the camera, based on empirical measurements and the concluding results show that camera principal output loss occurs in lighting and precipitation, which is calculated to increase performance errors by 50 percent. The authors in [166], outlined that rainfall is visible only in the near-field and has the characteristics of fog when far away. Besides, the authors propose the need for post-processing methods to mitigate the impact of rain and fog. For instance, a de-watering approach to enhance vision efficiency in rainfall is presented in [167]. We can hardly find studies which show the influence of snow and its interaction with the camera, due to the lack of snow-based simulators. Yet snow effects the mechanical operation of the camera when positioned outside the vehicle. For example, when there is moisture around the camera below the freezing point, thin layers of ice will cover the camera lens and prevent the viewer from seeing any movement, other than crystalline snow patterns. A similar effect was explained in [168], showing the difficulty of using data generated by cameras for lane detection, due to external factors, such as frost or droplets of moisture on the glass in front of cameras. If the camera is placed inside the windshield, then falling snow with varied shapes makes it difficult to trace and eliminate it from image processing, leading to image recognition problems of the target. Camera interaction with airborne particles was examined in [93], under controlled environmental conditions. The experiment was to test the camera’s perception performance in a controlled environment and the results highlight that the presence of particles (smoke and dust) influence the camera’s image quality and the contrast, leading to poor object classification. The advantages and limitations of the vision-based system are highlighted in Table 3. In Figure 9, the performance of the vision-based system is plotted for visualization, where the dashed red line presents the current state of the art.

### 4.6. Far-Infrared Camera

**Figure 10:**
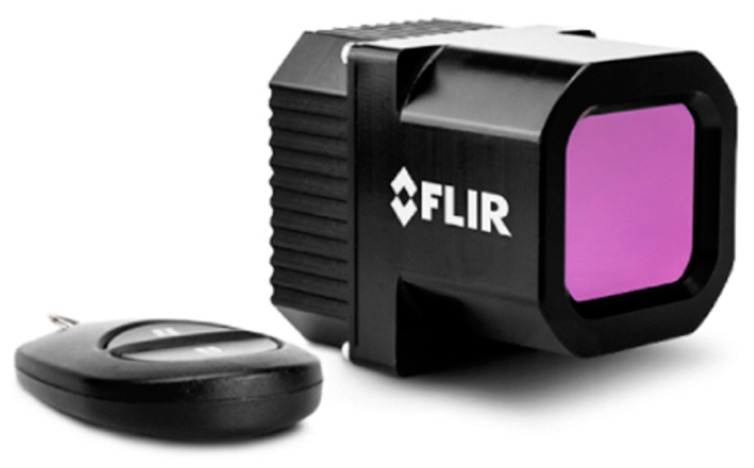
Thermal Vision FLIR ADK by FLIR Systems [170].

**Market penetration:** Following major improvements in vehicle lighting over the years, night driving and severe weather conditions are still difficult. According to the NHTSA statistics (discussed in the Introduction), night driving accidents account for one-third of all road accidents, and they account for half of the fatal accidents due to poor visibility [2]. Thermal imaging sensors provide additional advantages for existing night driving visible cameras. The far-infrared (FIR) camera is passive in design and consumes less energy than any other sensor. Currently, FLIR Systems is a noted manufacturer in the thermal camera field and has presented a dataset online for detection and tracking performance. In Figure 10, a thermal camera from FLIR Systems is shown and its technical specifications have been disclosed in [169].

**Working principle:** All objects emit infrared at temperatures above absolute 0 degrees, and this radiation increases with temperature. A long-infrared camera uses far-reaching infrared light waves to detect variations in natural heat (thermal radiation) emitted by objects. This description is subsequently translated into an image. The infrared spectrum varies between 0.8 μm and 1000 μm [171], and can be classified into near-infrared (NIR) ranges from 0.8 μm to 2.5 μm, mid-infrared (MIR) ranges from 2.5 μm to 25 μm, and far-infrared ranges from 25 μm to 1000 μm (also known as thermal infrared). A FIR camera detector is a focal plane array (FPA) with a resolution ranging from 160 × 120 to 1024 × 1024 pixels of micrometer-sized pixels, made of different infrared wavelength-sensitive materials. The FPA detector technology in infrared cameras is divided into thermal uncooled microbolometers and quantum detectors. An uncooled microbolometer is a common type of thermal detector made of metal or semiconductor materials and used more frequently in automotive applications. A brief overview of the state of the art of FIR cameras, such as construction design, operation, attenuation, and limitations, for interested audiences, can be found in [171]. For certain cases, because of the fundamental differences between visual and infrared imaging, the techniques used to detect pedestrians in the visible spectrum cannot be extended to infrared images, and other approaches must be used. In [172,173], fusion between the thermal camera and regular visible camera for the detection task is presented and the same comparison of detection techniques can be noted. In [174,175], thorough research on the detection of pedestrians using an FIR camera was presented. In addition, various techniques and algorithms, such as isolated ROI to extract targets from the image, followed by the classification of the extracted target and tracking, were outlined. Similarly, in [176,177,178,179,180,181,182,183,184,185,186,187,188,189], thermal camera studies have been shown to identify and track pedestrians, vehicles, and animals. 

**Figure 11:**
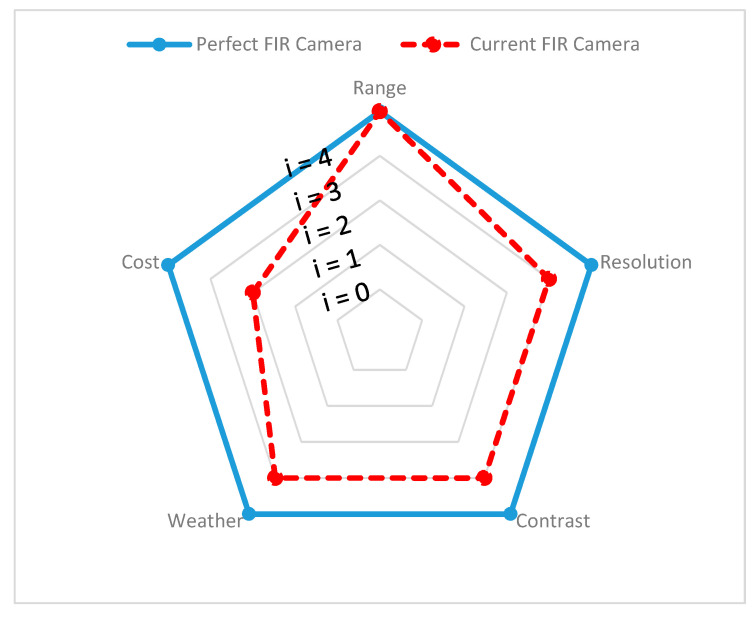
Far-infrared sensor spider chart. The difference between the red dashed line and the solid blue line is the performance gap.

**Performance reliability:** In comparison to the visible spectrum, FIR spectrum cameras lack a well-established public database and reliable benchmarking protocols for pedestrians, vehicle detection, classification, and tracking, making it difficult to test algorithms with a variety of well-known, typical automotive road scenes under various weather and lighting conditions. In general, FIR cameras operating in different environments undergo two atmospheric effects, absorption and dispersion. Atmospheric effects play critical role in preventing object radiation from entering the sensor. In automotive applications, FIR cameras are used to scan short distances of approximately 200–250 m and this short-distance sensing is not much influenced by atmospheric effects, compared to the aviation domain. In [190], a brief comparison in detection, classification, and recognition tasks in a normal and closed fog chamber with various vision spectral bands, such as visible (RGB), near-infrared (NIR), short-wave infrared (SWIR), and long-wave infrared (FIR), is presented and the results show that FIR spectral bands have superior performance capability, compared to other spectral bands in aerosol environments. Commercial manufacturers of thermal cameras claim that FIR thermal cameras are not affected by precipitate and airborne particles (fog, rain, snow, and dust), due to fact that air acts as a high-pass filter above 7.5μm [41], and due to their ability to penetrate different atmospheric conditions, they can be used to detect vehicles and obstacles robustly. The limitations and advantages of FIR are summarized in Table 3. Based on the information gathered, the performance of FIR systems is plotted on a spider chart in Figure 11, to visualize the performance gap.

### 4.7. Emerging Technology 

Due to the quality cost of trading, we limit our investigations to radar, lidar, cameras, and ultrasonic sensors. We need more robust technologies that can overcome existing technology requirements and perform multiple tasks with minimal problems in all weather conditions. In this sense, the European DENSE project [115], intends to develop a new sensor subsystem, although the article only talks about lidar sensor alternatives to the current state of the art, such as the use of a wavelength of 1550 nm instead of a wavelength of 905 nm to evaluate the performance in and of different climates. Very promising results with the use of 1550 nm have been presented, and there is still a need to explore studies that can improve sensor performance for robustness. In addition to lidar, studies on sub-camera systems, such as gated SWIR cameras proposed as a replacement for scanning lidar systems in real time, which handle back-scatter and provide dense depth at long ranges and reliable performance in climatic conditions, have been studied in the DENSE project. Similarly, in the field of localization, visual odometry [191], is a technology that is gaining popularity as a complementary GPS sensor in autonomous vehicles. Under the radar, investigations have been carried out on the ground penetration of signals that exhibit robustness in any road and weather condition [192].

## 5. Perspective on Sensor Fusion

Sensors are the key to the perception of the outside world in the automated driving system and whose cooperation performance directly determines the safety of automated driving vehicles. Some sensors may be redundant under some environmental conditions, and some may be complementary, assisting in successful cooperation to ensure consistent and accurate obstacle detection. Sensor fusion is the method of using multi-sensor information to calculate, recreate the environment, and generate dynamic device responses, resulting in a consistent and accurate representation of the vehicle’s surroundings and position for safer navigation. The study presented in [193], discusses the traditional limitations of sensor fusion and focuses on different strategies by demonstrating the effectiveness of combining various sensors with a model. Moreover, the advantages that come along with sensor fusion are highlighted therein. Sensor fusion architecture includes three different levels: (1) sensor level, where two or more detectors are merged into one hardware; (2) task level, where features are extracted and fused from each sensor; and (3) decision level, where the result is calculated by combining individual decisions. These three techniques are successful on their own. The result of a sensor fusion process is mostly a high-level representation and abstract with rich knowledge of the environment for successful semantic analysis. In varying weather conditions, sensor fusion is very vital. For instance, during heavy snowfall, detecting obstacles with lidar and cameras is highly uncertain and the aid of radar, FIR thermal cameras, and ultrasonic sensors can be combined to enhance the detection and multi-tracking of the target. However, to achieve most of the robustness using data fusion, one must be cautious about two vital strategies for enhancing vehicle navigation safely in all weather conditions, which are as follows:

- At the first stage, the fusion system must decide the features that make the navigation environment different from normal navigation. Therefore, there should be a context-aware mechanism that adapts the level of confidence of each piece of sensor information. When snow/rain is falling, the context-aware mechanism can simply use the camera and the weather data to confirm such an occurrence. Furthermore, a training process can be used to classify different weather-related road contexts.
- At the second stage, fusion processing can be carried out to provide the most recent sensing information and the corresponding level of confidence. Although the fusion concept can enhance the capability of the automated navigation feature in all weather conditions, the overall processing power can be considerable. Therefore, the fusion hardware and software architectures should be deeply analyzed before further implementation steps.

Table 4 provides guidance on an appropriate combination of sensors for ADAS driving safety applications (discussed in Section 3), which can greatly enhance the understanding of the robustness for all weather conditions. Table 4 is organized with ADAS applications on the left and multiple sensors across the top. In the following paragraph, we review sensor fusion strategies for driving safety applications and the proposal to resolve current drawbacks by suggesting and combining sensors with existing fusion techniques.

**Adaptive cruise control (ACC)**

Selecting appropriate sensors for adaptive cruise control (ACC) applications requires a sensor that can remotely detect and track obstacles at a distance in all weather conditions. The host vehicle lookahead at a distance is the key factor in stabilizing the vehicle in the lane to implement the ACC. A few sensor fusion methods are already available, used to collect long-range information in real time. For instance, in [194], the fusion advantages of lidar and radar are used to achieve the precision of spatial data like target velocity and distance to host vehicle. Work produces a real-time algorithm that lets an autonomous car quickly follow other cars at different speeds while maintaining a safe distance. In [195], a stereo camera and a far-infrared camera are analyzed and the results show that far-infrared images have been able to detect vehicles at long ranges but lack target classification, when compared to stereo vision. Fusing data from stereo and far-infrared cameras resulted in improved performance, reducing false positives in vehicle detection at long ranges and enhancing overall system performance. In [196], the optically passive far-infrared camera and optically active lidar was used in a multi-sensor detection system for railways, which successfully installed a test system of up to 400 m under normal conditions, ensuring long-distance safety at a speed of more than 120 km/h. Based on the above analysis, we suggest a long-range radar sensor and far-infrared thermal cameras for ACC systems, as both sensors have long-range capability and are minimally influenced by environmental changes. Lidar has also been suggested, as it meets the range criteria in the ACC application and users could select lidar for combination with other sensors, based upon sensitivity to varying climates and cost constraints. The remaining sensors such as cameras, ultrasonic, etc., lack long-range detections and are also influenced by the weather, which reduces their preference for use in ACC applications.

**Forward collision avoidance (FCA), traffic jam assist (TJA), and blind spot monitoring (BSM) systems**

For FCA, TJA, and BSM driving safety applications, long-range target detection and classification and motion tracking of targets are important requirements. The combination of lidar, vision, and radar offers reasonable coverage, a better classification, and long- to short-range motion tracking of targets, which enables the estimation of accurate distance and speed measurements of targets. Some of the current integration methods for three common sensors are as follows: in [197], a multi-modal fusion approach between radar and cameras is proposed. The proposed method is designed as a two-stage object detection network which uses radar detection and camera image features to estimate distance and to classify objects. The results of the experiment show that the proposed algorithm can accurately estimate the distance for all detected objects with a mean absolute error of 2.65 for all images captured. In [198], real-time experimental work is presented on the basis of cameras, lidar, and radar to achieve a high degree of object identification, classification, and tracking, in four weather conditions (such as cloudy and wet, bright day, night, rain and snow). The article demonstrated the efficiency of the three capable sensors with four different combinations (camera + radar, camera + lidar, lidar + radar, and radar + camera + lidar) based on a probabilistic algorithm. The results of the experiment show that for a full sensor set (radar + camera + lidar), in cloudy and sunny weather, objects are tracked with an accuracy of 98.5% and 99.8%, and the detected objects are classified with 87.5% accuracy. In night, rainy, and snowy weather conditions, the experimental results show that objects are reliably tracked at 98.9% and 99.5% accuracy with a classification score reduced to 74.4%. In our proposition, if an FIR thermal camera was used with a probabilistic algorithm proposed by the author in [198], with active toggling (switching) of the camera for varying climates, this could have enhanced the classification score in the experiment.

**Road and traffic sign recognition (TSR):**

For TSR safety applications, the classification and resolution of targets is an important factor. Vision-based solutions have always been superior in classification and recognition techniques. In [199], a robust technique to detect traffic lights during both day and night conditions and to measure the distance between the approaching vehicle and the traffic light is measured using a Bayesian filter. The results show that it was possible to detect traffic lights with 99.4% accuracy in the 10–115 m range. In [200], real-time lidar laser reflectivity and mono-camera color features were combined to detect and classify traffic signals; the fusion resulted in 95.87% detection and 95.07% classification with average computing accuracy. However, when faced with adverse weather, we can make use of an FIR thermal camera as an alternative, which can also provide classification and recognition information, as seen in [195].

**Lane departure warning and lane keeping warning (LDW and LKW) safety systems**

In LDW and LKW safety systems, a camera was used mainly as a means to distinguish between road and lane markings and, because of developments in algorithms, lidar and radar have the ability to search for clues in the environment related to the road and update tracking information. For instance, in [201], radar, along with a mono-camera, was used to detect road barriers and calculate lateral distance from the vehicle to the barrier, giving an estimate of vehicle position on the road. However, accuracy could not be achieved but when the results were compared with lidar tracking performance, it was concluded that radar was able to perform better even in the absence of lidar. Further studies on the improvement of radar detection and tracking of surrounding clues can help achieve robustness for various weather drives.

**Parking assistance (PA) systems**

In PA safety applications, obstacle detection and tracking at short distances are vital. In [202], data fusion between lidar and cameras is presented, which makes use of the SLAM algorithm to find parking spaces. The results of the experiment show the average recall and precision are 98% and 97%, respectively. However, the experiment was limited to stationary obstacle detection and rectangle parking slots. Similarly, in [203], an approach was presented to fuse cameras, ultrasonic sensors, and odometers to find a vacancy in the parking lot. Ultrasonic sensors were used to detect obstacles and later odometers and camera images were used for tracking obstacles and empty space location. The proposed method achieved 96.3% recall and 93.4% precision for different parking types, with a classification score of 97.5%. However, this approach is susceptible to weather change and the authors believe that the influence of varying weather may cause results to differ. As an alternative, if the system had short-range radar, this could boost the system robustness for all-weather parking. 

## 6. Conclusions

This article has provided a comprehensive review of the sensor technologies for both semi-autonomous and autonomous vehicles by considering the challenging issues related to their robustness in all weather conditions. A general view of the popular features of advanced driver assistance systems (ADASs) has been provided and the role of various sensors in these applications has been discussed with the limitations of these features in adverse climates. The study has provided a description of the advantages and disadvantages of the individual sensors, as well as their performance. Besides, the sensitivity of the different characteristics of the sensors, such as range, resolution, and speed detection in normal and difficult climatic conditions, has been discussed, and based on these characteristics, the differences in performance between current and ideal sensors for all weather conditions were mapped onto charts (known as spider charts). Our study has shown that the performance and robustness of the vehicle perception system can be enhanced by combining different sensors (such as cameras, radar, lidar, ultrasonic, and passive far-infrared cameras). For example, radar and passive infrared cameras provide a very good and effective range to detect obstacles in all weather conditions. However, lidar is not a widely favored sensor for the detection and tracking of obstacles due to disadvantages, such as cost and poor performance in rain, fog, and snow. On the other hand, cameras continue to play a key role in most automated navigation applications. The introduction of radar and passive infrared cameras improves the vehicle vision system in order to detect and monitor objects on the navigation scene. Nevertheless, the reliability of ADAS lateral features such as lane departure warning and lane keeping assistance, which rely on the visual road markings, cannot be easily improved under winter conditions. As a result, more studies are needed to solve this weakness. Nonetheless, when lateral driving assistance or parking assistance applications are carried out during heavy rainfall and snowfall, ultrasonic sensors combined with short-range radar can enable the vision system to succeed. Through identifying gaps in the research on semi-autonomous intelligent vehicles and shortcomings in current sensor technologies, this paper has thoroughly investigated the capabilities of current sensors for all weather drives. However, sensors and algorithms need to be made effective and reliable for any situation, and some points have been overlooked as far as the analysis is concerned, for example, the classification of bicycles, light poles, and pedestrians, which sometimes cause sensors to be mistaken when perceiving and, besides, the response time for slow-moving pedestrians and small animals or objects is of concern and, similarly, potholes and pitfalls that pose serious problems in adverse climates need to be addressed.

## Figures and Tables

**Table 1:** Indexes to interpret spider charts.

Criterion	Indexes (i)
Range	0—None 1—Very low performance 2—Low 3—High 4—Very high performance
Resolution	
Contrast	
Weather	
Cost	0—None
	1—Very high cost
	2—High cost
	3—Low cost
	4—Very low cost

**Table 2:** Ideal price selection of sensors from the actual market price.

Sensors	Actual Price	Ideal Price
Ultrasonic sensors	USD 16 to USD 40	USD 16 to USD 40
Automotive radar	USD 50 to USD 220	USD 50 to USD 220
Automotive lidar	USD 500 to USD 75,000	USD 100 to USD 10,000
Automotive mono-camera	USD 100 to USD 1000	USD 100 to USD 700
Automotive thermal camera	USD 700 to USD 3000	USD 100 to USD 1500

**Table 3:** List of advantages and disadvantages of perception sensors.

Sensor	Advantages	Disadvantages
Radar	The sensor makes it possible to see for long distances ahead of the car in poor visibility conditions, which can help avoid collisions. The sensor is small, lightweight, and affordable. The sensor requires less power than a lidar sensor since it has no moving parts. The sensor is more robust to failure compared to lidar. Radar is less expensive than lidar.	The obtained images have low accuracy and resolution. Information on detected objects is limited (such as neither precise shape nor color information). Increasing power may solve radar attenuation in a precipitate environment, but increasing power is not a viable economic solution. The mutual interference of radar sensors is a growing issue. The azimuthal and elevation resolution of automotive radars is poor, and this makes the detailed mapping of scenes and object classification difficult and error prone. The sensor cannot give a 360⁰ measurement of the surroundings.
Lidar	The sensor can see long distances ahead of the car in good visibility conditions (neither rain nor fog). The sensor can provide a full 360⁰ and 3D point clouds. The images have good accuracy and resolution. There are no significant interferences in multiple lidar sensors.	Lidar is more expensive than radar and camera. Transmission is sparse (not dense), due to which small objects (like wires and bars) remain undetected. Due to oscillating components, mechanical maintenance is high. When detecting wet surfaces, lidar shows poor discrimination of contrast compared to dry surfaces. The sensor requires more power than a radar sensor. The sensor is influenced by varying climatic conditions.
Ultrasonic	Ultrasonic sensors are useful for the detection of transparent objects and non-metal objects. Not influenced by varying climatic conditions. Low in cost and small in dimensions. At short ranges, higher resolution can be expected. Unlike cameras, ultrasonic sensors overcome pedestrian occlusion problems.	They are available for short-range distances only. Sensitive to temperatures and windy environments. Interference and reverberation are problematic when two ultrasonic sensors operate in two cars or are placed close together. Noise from environments may interfere with measurements.
Camera	Cameras maintain high resolution and color scales across the complete field of view. They offer a colorful perspective of the environment that helps to analyze the surroundings. Stereo cameras can provide a 3D geometry of objects. Cameras can robustly monitor and maintain information from surroundings over time. They are small in dimensions. Compared to lidar, they are cost-effective and easy to deploy on a vehicle.	The camera data require a powerful computation system to extract useful data. The sensor is sensitive to heavy rain, fog, and snowfall, which reduces the capability of the computer system to reliably interpret the surrounding scene. The distance to obstacle accuracy is limited.
Far-Infrared	Far-infrared (FIR) camera images depend on the target temperature and radiated heat. Therefore, light conditions and object surface features do not influence them. Compared to lidar, FIR sensors are cheaper and smaller. They have improved situational awareness at night. FIR sensing range can cover up to 200 m or more horizontally and detect possible hazards ahead. They have a better vision through dust, fog, and snow compared to cameras.	FIR camera data require demanding computation sources and robust algorithms to extract useful data. This sensor is expensive, compared to Charging Coupling Device (CCD) or the Complementary Metal Oxide Semiconductor (CMOS) cameras. The resolution of the FIR camera is low in comparison to the visible camera and provides images in grayscale. Due to this, fast-changing moments of objects are quite challenging to detect and classify in real time. Since FIR systems calculate based on temperature differences, it is often difficult to distinguish between specific targets of interest in cold climate scenarios. Partial occlusion of the target causes classifiers to ignore the target (like a pedestrian standing behind a car or a group of pedestrians overlapping each other). Solutions to overcome this problem have been studied and, besides, they cannot provide information about the distance to obstacles.

**Table 4:** A possible combination of sensors for all-weather navigation.

Application	Radar			Ultrasonic	Lidar			Camera		Far-Infrared
	Short Range	Medium Range	Long Range		Short Range	Medium Range	Long Range	Monocular Camera	Stereo Camera	
Adaptive Cruise Control			√				√			√
Forward Collision Avoidance		√	√			√		√	√	√
Road/Traffic Sign Recognition						√		√	√	√
Traffic Jam Assist	√	√			√	√		√	√	√
Lane Departure and Lane Keeping Assistance		√				√		√	√	
Blind Spot Monitoring	√	√		√	√	√		√	√	
Parking Assistance	√	√		√	√	√		√	√	
